# DNA methylation may partly explain psychotropic drug-induced metabolic side effects: results from a prospective 1-month observational study

**DOI:** 10.1186/s13148-024-01648-4

**Published:** 2024-02-28

**Authors:** Céline Dubath, Eleonora Porcu, Aurélie Delacrétaz, Claire Grosu, Nermine Laaboub, Marianna Piras, Armin von Gunten, Philippe Conus, Kerstin Jessica Plessen, Zoltán Kutalik, Chin Bin Eap

**Affiliations:** 1 Unit of Pharmacogenetics and Clinical Psychopharmacology, Center for Psychiatric Neuroscience, Department of Psychiatry, Lausanne University Hospital, University of Lausanne, Hôpital de Cery, 1008 Prilly, Lausanne, Switzerland; 2 Swiss Institute of Bioinformatics, Lausanne, Switzerland; 3 Center for Integrative Genomics, University of Lausanne, Lausanne, Switzerland; 4 Service of Old Age Psychiatry, Department of Psychiatry, Lausanne University Hospital, University of Lausanne, Prilly, Switzerland; 5 Service of General Psychiatry, Department of Psychiatry, Lausanne University Hospital, University of Lausanne, Prilly, Switzerland; 6 Service of Child and Adolescent Psychiatry, Department of Psychiatry, Lausanne University Hospital, University of Lausanne, Lausanne, Switzerland; 7 Department of Computational Biology, University of Lausanne, Lausanne, Switzerland; 8 University Center for Primary Care and Public Health, University of Lausanne, Lausanne, Switzerland; 9 School of Pharmaceutical Sciences, University of Geneva, University of Lausanne, Geneva, Switzerland; 10 Center for Research and Innovation in Clinical Pharmaceutical Sciences, University of Lausanne, Lausanne, Switzerland; 11 Institute of Pharmaceutical Sciences of Western Switzerland, University of Geneva, University of Lausanne, Geneva, Switzerland

**Keywords:** DNA methylation, Metabolic side effects, Psychotropic drugs, EWAS

## Abstract

**Background:**

Metabolic side effects of psychotropic medications are a major drawback to patients’ successful treatment. Using an epigenome-wide approach, we aimed to investigate DNA methylation changes occurring secondary to psychotropic treatment and evaluate associations between 1-month metabolic changes and both baseline and 1-month changes in DNA methylation levels. Seventy-nine patients starting a weight gain inducing psychotropic treatment were selected from the PsyMetab study cohort. Epigenome-wide DNA methylation was measured at baseline and after 1 month of treatment, using the Illumina Methylation EPIC BeadChip.

**Results:**

A global methylation increase was noted after the first month of treatment, which was more pronounced (*p* < 2.2 × 10^–16^) in patients whose weight remained stable (< 2.5% weight increase). Epigenome-wide significant methylation changes (*p* < 9 × 10^−8^) were observed at 52 loci in the whole cohort. When restricting the analysis to patients who underwent important early weight gain (≥ 5% weight increase), one locus (cg12209987) showed a significant increase in methylation levels (*p* = 3.8 × 10^–8^), which was also associated with increased weight gain in the whole cohort (*p* = 0.004). Epigenome-wide association analyses failed to identify a significant link between metabolic changes and methylation data. Nevertheless, among the strongest associations, a potential causal effect of the baseline methylation level of cg11622362 on glycemia was revealed by a two-sample Mendelian randomization analysis (*n* = 3841 for instrument-exposure association; *n* = 314,916 for instrument-outcome association).

**Conclusion:**

These findings provide new insights into the mechanisms of psychotropic drug-induced weight gain, revealing important epigenetic alterations upon treatment, some of which may play a mediatory role.

**Supplementary Information:**

The online version contains supplementary material available at 10.1186/s13148-024-01648-4.

## Background

Psychiatric disorders including schizophrenia, bipolar and major depression disorders are associated with a high prevalence of cardiovascular diseases (CVDs) leading to premature death [1]. This excessive cardiovascular risk results from a combination of factors including psychiatric disease-related aspects, shared genetic susceptibilities, unhealthy lifestyle and adverse effects of treatment. Psychotropic medications, such as antipsychotics (most atypical and some typical), mood stabilizers (i.e. lithium and valproate) and some antidepressants (i.e. mirtazapine) can indeed increase the risk of metabolic disorders including obesity, dyslipidemia, type 2 diabetes and hypertension [2, 3]. Although weight gain and obesity are major risk factors for the development of other metabolic abnormalities, dysregulations following psychotropic treatment have also been observed without or with only slight weight gain, suggesting the implication of other mechanisms. Psychotropic drug-induced disturbances in lipid and glucose levels may thus occur following an increase in adiposity and body weight, but also through independent pathways [2, 4, 5]. The multifactorial mechanisms underlying the development of these adverse effects are only partially understood, and epigenetic changes, driven by both environmental and genetic factors, may contribute to explaining their occurrence. Administration of psychotropic drugs may induce alterations in DNA methylation, profoundly influencing gene regulation and expression [6–9]. Although this molecular mechanism is currently extensively studied in relation to treatment response, the pharmacoepigenetic of psychotropic drug-induced metabolic side effects remains underexplored [10–14]. In the general population, some differentially methylated sites within the genome have already been reported to be causally linked to CVDs [15]. It is thus likely that psychotropic drugs also act through epigenetic mechanisms to increase CVDs risks.

Global methylation in relation to atypical antipsychotic treatment and metabolic parameters was assessed in three different studies [16–18]. While the first study yielded no conclusive results [16], the second study showed that atypical antipsychotic use and insulin resistance were both significantly associated with lower global methylation [17], and the last study highlighted a positive correlation between methylation levels and insulin resistance [18]. These mixed preliminary results point towards an effect of psychotropic drugs on global methylation levels leading to metabolic side effects.

In a candidate gene approach, epigenetic analyses focusing on genes or genetic pathways with a highly probable role in the development of metabolic syndrome (MetS) induced by psychotropic medications were performed. DNA methylation of the catechol-O-methyl transferase (*COMT*) gene [19] and insulin growth factor 2 (*IGF2*) gene [20] was measured, but no significant relationships between epigenetic variability and metabolic parameters were found. Nonetheless, an association between changes in the methylation level of the CREB-regulated transcription coactivator 1 (*CRTC1*) gene and early weight gain following psychotropic treatment initiation was shown [21], and a positive trend for increased methylation of protein kinase B (*AKT2)* associated with insulin resistance was observed in patients treated with atypical antipsychotics, while the opposite correlation was revealed in mood stabilizer users [22].

Hypothesis-driven studies may help reveal how modulation of genes leads to metabolic side effects, but given the mixed results obtained to date, they might fail to capture the complex effects of psychotropic drugs in targeting only specific sites. To overcome this limitation, epigenome-wide association studies (EWAS) may help to further investigate the role of epigenetics in psychotropic drug-induced metabolic side effects. The only studies that used this approach so far allowed to discover a differentially methylated site in the fatty acyl CoA reductase 2 (*FAR2*) gene that was associated with insulin resistance [23] and another site in the cadherin-like 22 (*CDH22*) gene that was associated with MetS [24].

Within the current study, we aimed to use the same hypothesis-free strategy to explore global methylation associated with metabolic alterations, in combination with a hypothesis-driven approach, addressing the relationship between site- or gene-specific DNA methylation patterns and metabolic side effects. Unlike previous cross-sectional studies that highlighted associations present in samples of patients at a given time, we wished to capture the effects of psychotropic drugs occurring with treatment onset and followed a longitudinal design, analysing samples collected at the start of treatment and after one month. We then sought to investigate whether baseline methylation or changes in methylation were associated with changes in weight and metabolic parameters.

## Methods

### Study design

Patients were recruited at the Department of Psychiatry of the Lausanne University Hospital as part of a large observational cohort study (PsyMetab) described elsewhere [25]. Briefly, patients were recruited in PsyMetab when starting a treatment with a psychotropic drug known to induce weight gain and metabolic alterations (including most antipsychotics, mood stabilizers and some antidepressants). They were then followed-up in compliance with a local clinical guideline to control the occurrence of side effects. From this cohort, 79 patients with informed consent, included between February 2008 and February 2016, were selected, as illustrated in the flow chart available in the Additional file 1: Fig. S1. Most patients were not drug naïve and had already received psychotropic medications before entering the study. Early weight gain is a good predictor of metabolic complications following treatment introduction [26, 27]. Patients with a ≥ 5% weight increase in one month show important long-term weight gain (≥ 15% after 3 months; ≥ 20% after 12 months), and the reasons for this rapid and consistent weight gain are only poorly understood. We thus chose to include patients with important early weight gain (*n* = 39) and patients with no or minimal weight gain (*n* = 40). A comparative table (Additional file 1: Table S1) with the main characteristics of selected vs unselected patients is provided to ensure that the sample is representative of the whole cohort.

When available, metabolic parameters including blood pressure, plasma levels of glucose, triglycerides, and cholesterol (total cholesterol, LDL cholesterol and HDL cholesterol), were retrieved from medical files. Metabolic syndrome status was assessed using the International Diabetes Federation (IDF) definition [28]. Information on diagnosis, age at medication onset, smoking status and sex was also extracted from medical records and/or specific questionnaires. Diagnostic groups were established according to ICD-10 classification, and psychotropic medications were categorized according to their propensity to induce weight gain in three groups, as already defined in previous analyses involving the PsyMetab cohort [21, 29–31], i.e., low-risk (e.g., amisulpride *n* = 3, aripiprazole *n* = 10); medium-risk (e.g., quetiapine *n* = 23, risperidone *n* = 8, lithium *n* = 8, and mirtazapine *n* = 3), and high-risk (e.g., valproate *n* = 1, olanzapine *n* = 19, and clozapine *n* = 4) [32–35].

### DNA methylation

Blood samples were collected for each patient at the start of treatment (T0) and after one month (T1). Genomic DNA was obtained from whole blood as previously described [21]. DNA methylation was analysed at the iGE3 genomics platform of the University of Geneva (Home - iGE3 Genomics Platform - UNIGE) using the Illumina Infinium Methylation EPIC BeadChip, enabling the measurement of over 850,000 methylation sites (Illumina, San Diego, CA, USA).

### Statistical analysis

Demographic and clinical characteristics of patients were described and compared between patients who gained 5% or more of their initial weight (considered cases) and patients whose weight remained stable (considered controls) using Wilcoxon Mann–Whitney rank-sum and Pearson *χ*^2^ tests for continuous and categorical variables, respectively.

All following statistical analyses were conducted using M-values to estimate methylation levels as this metric shows good statistical validity, and significant results were illustrated using β-values as it enables better interpretability [36]. Principal component analysis was performed on M-values, and the top three principal components were used as covariates in regression analyses to capture unmeasured confounding effects. Additional multivariable models adjusting for cell type composition were also conducted as sensitivity analyses, i.e., estimating the proportion of cell subtypes using the EpiDISH (Epigenetic Dissection of Intra-Sample Heterogeneity) algorithm [37] and alternatively using percent neutrophils measured in blood samples in a subset of patients with available data. All analyses were 2-sided with alpha = 0.05. Data preparation was conducted using Stata 16 (StataCorp; College Station, Texas), and analyses were performed using the R environment for statistical computing version 4.0.2.

#### Global and epigenome-wide methylation changes and their association with weight change

Global DNA methylation levels were estimated using the mean methylation level of all the analyzed sites, and differences between T0 and T1 were evaluated using paired Student’s *t*-tests, once in all participants and separately in cases and controls.

To identify loci with epigenome-wide significant T0–T1 changes in the entire cohort, and again, separately according to early weight gain groups, paired Student’s* t*-tests were performed, not adjusting on additional variables. The family-wise error rate (FWER) was controlled by the Bonferroni correction, and hence, nominal P values passing the 9 × 10^–8^ threshold (alternatively 4.5 × 10^–8^ for the stratified analyses) were considered statistically significant [38]. The association between the CpG sites with epigenome-wide significant T0-T1 changes and weight gain was evaluated using a linear model. Weight change was adjusted for baseline BMI, smoking status, sex, age, treatment propensity to induce weight gain (categorized as low, moderate, or high as described above) and the first three principal components of methylation data (or cellular composition, in sensitivity analyses) and normalized using an inverse normal quantile transformation (INQT).

#### Epigenome-wide association analyses (EWAS) with metabolic phenotypes in PsyMetab and investigation of causality by Mendelian randomization

To identify CpG sites with baseline methylation levels or changes in methylation levels (between T0 and T1) associated with increased body weight gain, linear regressions were performed, using the same linear model as described above. Similarly, to identify CpG sites associated with changes in glucose and lipid plasma levels (fewer patients with available data: glucose (*n* = 25), triglycerides (*n* = 39) and total- (*n* = 38), HDL- (*n* = 38) and LDL-cholesterol (*n* = 37)), linear regression models were run, adjusting for covariates of baseline phenotype, smoking status, sex, age, treatment propensity to induce weight gain and the first three principal components of methylation data as well as for the presence of a treatment for diabetes or dyslipidemia for glucose and lipid phenotypes, respectively. For all EWAS, phenotypic traits were normalized, using an INQT. FWER was again controlled by the Bonferroni correction, with nominal P values below 9 × 10^–8^ considered statistically significant [38].

We selected CpG sites among the top 10 most significant associations with each metabolic phenotype and estimated their causal effect by Mendelian randomization (MR) in independent study samples. MR methodology has been presented in depth elsewhere [39]. Briefly, the random distribution of single nucleotide polymorphisms (SNPs) at birth reduces the possibility of reverse causation or confounding as explanations for the association between the exposure and outcome in the same way that the allocation of an intervention in a randomized controlled trial minimizes this possibility. Methylation quantitative trait loci (*cis*-meQTLs), discovered by Bonder et al. in a cohort of 3841 European individuals [40], were used as instrumental variables and CpGs linked to a minimum of two SNPs were retained. The association of these genetic variants with the metabolic phenotypes were then derived from genome wide association studies (GWAS) performed in the UKBiobank (UKB), selecting British unrelated individuals (http://www.nealelab.is/uk-biobank, *n *≈ 300,000, depending on the phenotype). The details of the UKB have been described elsewhere [41]. Briefly, UKB is a prospective cohort study including more than 500,000 individuals (40–69 years) recruited from the United Kingdom during 2006–2010. Two-sample MR analyses, using the inverse-variance weighted method, were eventually performed to estimate the causal relationships between CpGs and related metabolic phenotypes [42].

#### Validation of previous findings

The role of methylation patterns in the three genes that have already been associated with metabolic outcomes in psychotropic drug treated patients was further characterized with our data. Thus, the associations between the average methylation in the region of the candidate gene *AKT2* (5 out of 22 CpG sites present in our data) [22] and 1-month glucose change were evaluated; the association between 1-month glucose change and the EWAS hit with insulin resistance [23], namely, cg10171063 located in *FAR2*, was assessed; and the association between the EWAS hit with MetS [24], namely, cg04640913 located in *CDH22*, and MetS was eventually tested. Linear and logistic models, adjusting for baseline phenotype, smoking status, sex, age, treatment propensity to induce weight gain and the first three principal components of methylation data, were used for these analyses.

#### Hypothesis-driven analyses

Three hypothesis-driven analyses were performed to select specific subgroups of CpG sites with a putative key role in weight gain or in psychotropic drug-induced metabolic side effects. The associations between baseline or T0-T1 change in methylation level with the metabolic phenotypes in the psychiatric cohort were assessed using the same models as described above. Associations with Bonferroni-corrected P values below 0.05 were considered statistically significant. These three analyses are further described in the Additional file 1.

## Results

### Population characteristics

The clinical and demographic parameters of the 79 included participants are presented in Table 1. The median age of the cohort was 37 years (range = 16–84) and men represented 50.6% of the patients. The proportion of smokers was 35.9% in patients with early weight gain, while it reached 65.0% in patients whose weight remained stable during the first month of treatment (*p* = 0.01). Patients suffered mainly from psychotic disorders (45.6%), followed by bipolar disorder (22.8%), schizoaffective disorder (12.7%) and depressive disorders (10.1%), with no difference in relation to early weight gain status (*p* = 0.33). They were treated primarily with psychotropic drugs carrying an intermediate propensity to induce metabolic side effects (53.2%), while 30.4% and 16.5% of participants received a treatment with a high and low risk, respectively. The median BMI at treatment initiation was 23.1 kg/m^2^ (range = 15.2–37.5) and it did not differ according to early weight gain status (*p* = 0.16). Consistent with the study design and participant selection, the weight gain difference across both patient groups during the study period was statistically significant (*p* < 10^–4^), and interestingly, a significant increase in MetS prevalence was noted among cases (*p* = 0.05).

**Table 1:** Clinical and demographic parameters of the study sample, with global methylation change in participants stratified according to early weight gain status

	*N*	Total sample	Controls^a^ (*n* = 40)	Cases^a^ (*n* = 39)	*p*-value^b^
Age, median (range), *y*	79	37 (16–84)	37 (17–84)	39 (16–83)	0.56
Men, *n* (%)	79	40 (50.6)	20 (50.0)	20 (51.3)	0.9
Smoking, *n* (%)	79	40 (50.6)	26 (65.0)	14 (35.9)	**0.01**
Main diagnosis, *n* (%)	79				0.33
Psychotic disorders (F20-F24; F28-F29)		36 (45.6)	17 (42.5)	19 (48.7)	
Schizoaffective disorders (F25)		10 (12.7)	6 (15.0)	4 (10.3)	
Bipolar disorders (F30–F31)		18 (22.8)	12 (30.0)	6 (15.4)	
Depressive disorders (F32–F33)		8 (10.1)	2 (5.0)	6 (15.4)	
Other		7 (8.9)	3 (7.5)	4 (10.3)	
Psychotropic treatment group, *n* (%)^c^	79				0.74
Low risk of WG		13 (16.5)	6 (15.0)	7 (18.0)	
Medium risk of WG		42 (53.2)	23 (57.5)	19 (48.7)	
High risk of WG		24 (30.4)	11 (27.5)	13 (33.3)	
BMI, median (range), kg/m^2^	79				
Baseline		23.1 (15.2–37.5)	23.5 (17.1–36.5)	21.9 (15.2–37.5)	0.16
First month		23.9 (17.0–39.5)	23.8 (17.1–36.5)	24.1 (17.0–39.5)	0.81
*p*-value^d^		** < 10**^**–4**^	** < 10**^**–4**^	** < 10**^**–4**^	
WG, median (range), %		2.4 (0–23.0)	0.5 (0–2.4)	7 (5.2–23.0)	** < 10**^**–4**^
Metabolic syndrome prevalence, *n* (%)^e^	51				
Baseline		3 (5.9)	3 (11.1)	0 (0.0)	0.09
First month		8 (15.7)	4 (14.8)	4 (16.7)	0.86
*p*-value^d^		0.23	0.32	**0.05**	
Global baseline (T0) methylation (*β*-value), mean (range), %	79	61.78 (58.52–64.01)	61.80 (58.52–64.01)	61.77 (59.20–63.86)	0.87
Global methylation (*β*-value) increase (*T*1–*T*0), mean (95%CI), %	79	0.187 (0.185–0.190)	0.201 (0.198–0.204)	0.174 (0.170–0.177)	** < 2.2 × 10**^**–16**^

*BMI* body mass index, *WG* weight gain

^a^Patients who gained 5% or more of their initial weight were considered cases, and patients whose weight remained stable were considered controls

^b^Statistical significance for differences between groups was tested using the Wilcoxon Mann–Whitney rank-sum test for continuous variables (except for the differences in methylation levels which were assessed using Student’s *t*-test) and Pearson *χ*^2^ test of independence for categorical variables. Significant *p*-values (< 0.05) are indicated in bold

^c^Psychotropic drugs are considered to confer a low, medium and high risk of metabolic side effects for amisulpride and aripiprazole; risperidone, quetiapine, mirtazapine and lithium; and valproate, clozapine and olanzapine, respectively

^d^Statistical significance for differences between baseline and 1-month values was tested using the Wilcoxon signed rank test for matched pairs for continuous variables and McNemar test for categorical variables. Significant *p* values (< 0.05) are indicated in bold

^e^Metabolic syndrome was evaluated according to the definition of the International Diabetes Federation [28]

### Global and epigenome-wide methylation changes and their association with weight change

The global mean methylation level was higher one month after treatment initiation (Table 1), and this increase was greater in patients whose weight remained stable (*p* < 2.2 × 10^–16^).

A significant change between baseline and 1-month methylation levels (*p* < 9 × 10^−8^) was observed in 52 methylation sites in the entire cohort. The complete list of CpG sites and their methylation levels is available in Table 2, with information related to the genomic location of these sites. The change in methylation level at these 52 CpG sites was not associated with weight gain following treatment onset (nominal *p* > 0.09, data not shown).

**Table 2:** Baseline and 1-month methylation levels of 52 methylation sites with significant changes following treatment initiation

CpG site	Baseline methylation level *(β-value)*, median (IQR), %	1-month methylation level *(β-value)*, median (IQR), %	*p*-value^a^	CHR	CpG position^b^	Reference gene^c^	Location of CpG related to gene^c^	Relation to CpG Island^d^
*cg10992198*	67.7 (64.3–71.5)	70.7 (66.2–73.1)	0.00005	19	36,552,038	WDR62*	Body	
*cg12120973*	68.3 (65.1–71.7)	70.0 (67.1–74.3)	0.00009	1	1,215,925	SCNN1D*	5'UTR	
*cg05034501*	84.6 (82.9–86.4)	87.1 (85.1–89.5)	0.0001	3	148,985,369			
*cg05235884*	81.3 (80.2–84.1)	82.8 (80.9–84.8)	0.0001	6	30,131,806	*TRIM15*	1stExon	
*cg11678481*	75.6 (73.9–77.8)	77.8 (75.6–78.9)	0.0001	1	151,682,882	CELF3*	Body	
*cg13422535*	61.9 (58.9–64.1)	62.2 (60.2–65.6)	0.0001	22	47,077,682			S_Shelf
*cg22329201*	76.1 (73.1–77.4)	77.1 (74.8–79.3)	0.0002	4	3,569,189			S_Shelf
*cg20548564*	89.4 (88.3–90.6)	90.5 (89.3–91.3)	0.0004	13	28,240,073	*POLR1D*	Body	
*cg20626144*	74.0 (72.0–76.1)	75.1 (73.9–77.3)	0.0005	17	157,107	*RPH3AL*	Body	Island
*cg24357026*	9.8 (7.7–11.4)	8.3 (6.7–9.9)	0.0005	19	36,705,589	ZNF146*	5'UTR	Island
*cg02644728*	74.1 (71.8–76.7)	75.6 (73.7–77.9)	0.0005	11	83,324,374	DLG2*	Body	
*cg02692850*	65.5 (63.0–67.3)	66.4 (64.3–67.9)	0.0006	21	44,607,862			
*cg05129295*	56.4 (54.2–58.7)	58.4 (55.7–60.4)	0.0007	8	1,316,294			
*cg05696006*	79.0 (76.5–79.9)	79.9 (77.9–81.4)	0.0007	19	7,622,906	PNPLA6*	Body	S_Shore
*cg15223933*	74.5 (72.9–76.1)	75.5 (74.1–77.0)	0.0008	17	39,916,203	JUP*	Body	S_Shelf
*cg13691093*	87.4 (84.9–89.2)	89.0 (86.9–91.0)	0.002	8	2,031,672	*MYOM2*	Body	
*cg27452651*	7.1 (6.1–8.1)	6.2 (5.4–6.9)	0.002	22	50,312,357	ALG12*	TSS1500	Island
*cg13982468*	88.8 (87.3–89.6)	89.8 (88.9–90.6)	0.004	12	120,571,393	*GCN1*	Body	
*cg22800959*	94.6 (93.6–95.3)	95.2 (94.4–95.9)	0.004	6	32,020,477	*TNXB*	Body	
*cg11702503*	82.2 (80.3–84.0)	83.3 (81.6–85.0)	0.004	19	6,215,254	*MLLT1*	Body	Island
*cg20710898*	69.8 (68.5–71.4)	71.4 (69.7–73.2)	0.004	10	96,996,833			
*cg23628099*	68.2 (64.7–72.0)	70.0 (67.3–73.6)	0.004	8	48,089,762			
*cg25226092*	63.2 (61.4–64.6)	64.3 (62.4–66.3)	0.005	3	39,508,863	MOBP*	TSS1500	
*cg07769732*	65.3 (61.9–67.7)	67.0 (62.9–70.3)	0.005	2	8,815,465			N_Shore
*cg10825881*	77.7 (75.7–79.0)	78.5 (77.4–80.0)	0.005	15	52,202,509	*TMOD3*	3'UTR	
*cg17866025*	62.4 (59.7–64.5)	64.0 (61.6–65.5)	0.006	1	39,051,682			
*cg18930928*	79.2 (77.7–81.2)	80.2 (78.6–82.1)	0.006	1	205,210,953	*TMCC2*	Body	S_Shore
*cg02624558*	5.2 (4.3–6.7)	4.9 (3.6–5.8)	0.008	1	202,777,611	*KDM5B*	TSS200	Island
*cg05103574*	80.3 (78.6–82.0)	81.4 (79.9–83.2)	0.008	1	29,527,219	MECR*	Body	
*cg08243790*	64.1 (62.1–65.9)	65.4 (63.8–67.3)	0.008	8	127,485,353			
*cg14885690*	78.4 (76.2–80.3)	79.9 (77.8–82.4)	0.009	1	43,195,659			
*cg27275821*	75.5 (72.8–78.0)	78.6 (76.0–80.5)	0.009	1	89,144,331	*PKN2-AS1*	Body	
*cg00661205*	75.6 (74.4–77.6)	77.0 (75.6–78.3)	0.01	13	42,400,902	KIAA0564*	Body	
*cg08581040*	83.1 (80.4–85.0)	84.5 (82.5–86.6)	0.01	4	39,172,577			S_Shore
*cg09518293*	71.0 (68.6–73.1)	72.2 (70.8–74.9)	0.01	2	179,673,636	TTN*	TSS1500	
*cg10231096*	85.8 (82.7–89.2)	87.5 (84.3–90.5)	0.01	15	31,451,780	*TRPM1*	Body	
*cg13003350*	81.2 (78.4–83.7)	83.2 (80.0–85.2)	0.01	6	27,830,500			N_Shelf
*cg13091133*	67.4 (65.9–70.1)	69.4 (66.7–71.7)	0.01	4	42,190,340			
*cg16106297*	75.2 (73.2–77.0)	76.3 (74.5–77.8)	0.01	19	58,556,609	*ZSCAN1*	Body	Island
*cg24870895*	76.0 (74.1–77.6)	77.6 (75.3–78.9)	0.01	15	40,768,542			
*cg26032412*	52.5 (51.0–54.1)	53.8 (51.6–55.5)	0.01	10	130,300,419			S_Shore
*cg01823005*	79.0 (75.8–82.6)	82.0 (78.3–84.1)	0.02	10	90,691,868	*ACTA2-AS1*	TSS1500	
*cg03797660*	89.6 (88.1–91.1)	90.6 (89.7–91.9)	0.02	16	89,035,228	*CBFA2T3*	Body	S_Shore
*cg04043710*	67.6 (64.0–70.4)	69.4 (65.9–71.0)	0.02	16	9,448,891			
*cg05864168*	89.7 (88.8–91.0)	90.8 (89.6–91.8)	0.02	1	185,110,088	TRMT1L*	Body	
*cg11867718*	70.7 (68.5–72.9)	72.1 (69.8–73.7)	0.02	11	61,647,697	*FADS3*	Body	
*cg19267144*	76.5 (75.2–78.5)	77.8 (76.2–79.6)	0.02	17	40,956,947	*CNTD1*	Body	
*cg20073412*	95.5 (94.9–96.3)	96.3 (95.6–97.0)	0.02	11	131,560,558	*NTM*	Body	Island
*cg23313005*	82.7 (81.6–84.2)	84.0 (82.7–85.4)	0.02	5	176,965,046	FAM193B*	Body	
*cg25470611*	74.8 (71.4–78.0)	76.3 (72.9–79.7)	0.02	11	71,121,630	*FLJ42102*	Body	
*cg26856604*	74.4 (71.0–76.7)	75.8 (73.6–77.8)	0.02	1	10,370,540	*KIF1B*	Body	
*cg05058976*	86.1 (84.5–87.3)	87.1 (85.6–88.3)	0.03	16	3,637,956	*BTBD12*	Body	N_Shore

*CHR* chromosome, *IQR* interquartile range

^a^Statistical significance for differences between baseline and 1-month methylation was tested using paired Student’s* t*-test, without adjustment on additional factors. Bonferroni correction for epigenome-wide analyses was applied

^b^CpG positions refer to Genome Research Consortium human genome build 37 (GRCh37)/UCSC human genome 19 (hg19)

^c^Reference genes for the methylation sites, and gene regions where the CpGs are located according to the UCSC database. Empty fields indicate an intergenic location

^*^Specifies there exists > 1 gene or gene transcript at this location. TSS200 = 0–200 bases upstream of the transcriptional start site (TSS); TSS1500 = 200–1500 bases upstream of the TSS; 5′UTR = within the 5′ untranslated region, between the TSS and the ATG start site; Body = Between the ATG and stop codon, irrespective of the presence of introns, exons, TSS, or promoters; 3′UTR = between the stop codon and poly A signal

^d^The relation to a putative nearby CpG island, according to the UCSC database, is given. Shore = 0–2 kb from island; Shelf = 2–4 kb from island; N = upstream (5′) of CpG island; S = downstream (3′) of CpG island

**Fig. 1:**
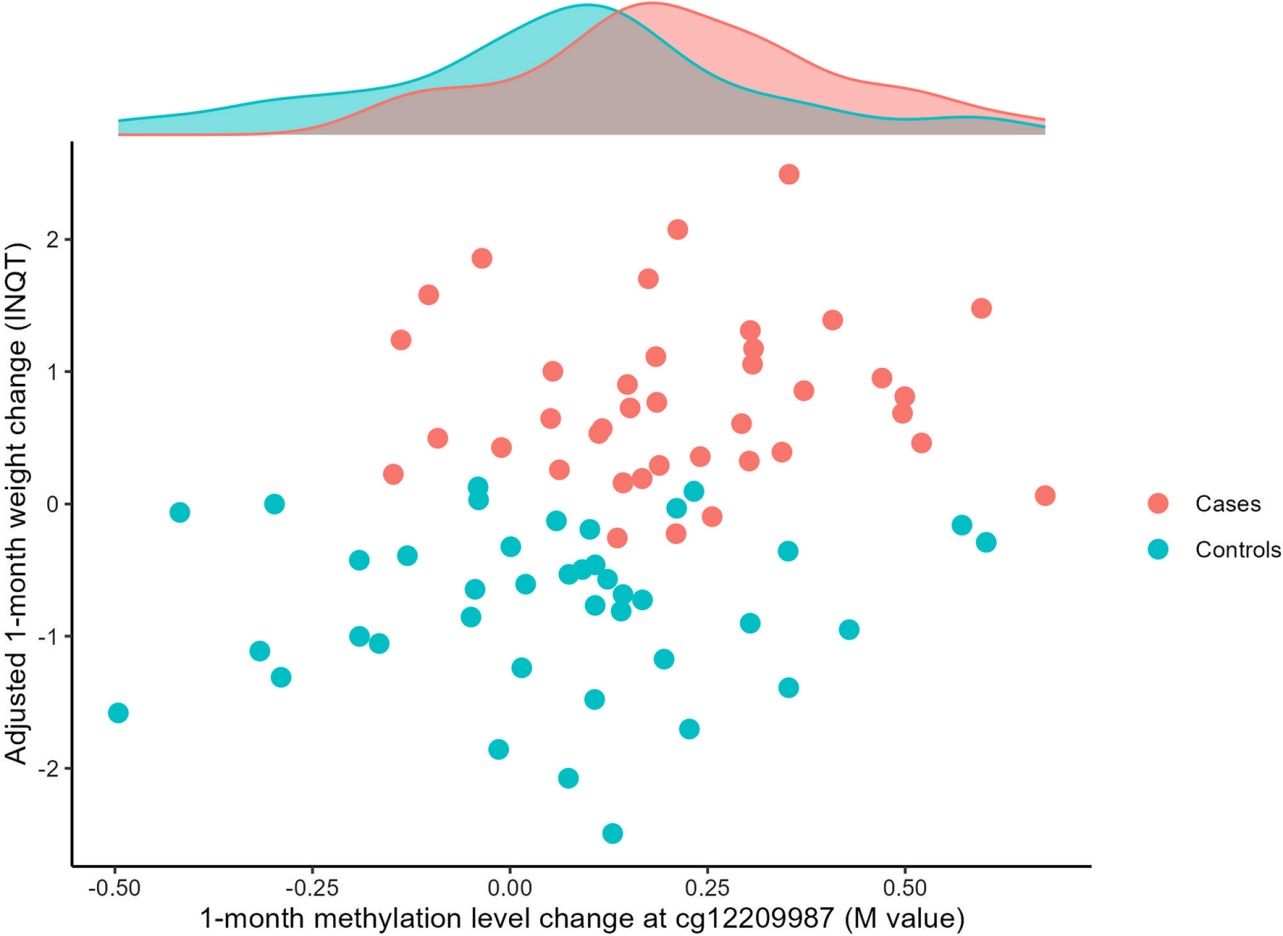
Association between 1-month methylation level change at cg12209987 and weight change. Weight change was adjusted for baseline BMI, smoking status, sex, age, treatment propensity to induce weight gain and the first three principal components of methylation data, and normalized using an inverse normal quantile transformation (INQT). The methylation level change was assessed using the *M*-value metric. Patients who gained 5% or more of their initial weight were considered cases, and patients whose weight remained stable were considered controls

The stratified analyses, restricted to samples with early weight gain, revealed a significant change (between T0 and T1) in methylation at one specific locus, namely cg12209987. This methylation site is located on chromosome 5, in an intergenic region 60 kb upstream of the *PSMC1P5* pseudogene. Its median methylation level increased from 69.7% (interquartile range [IQR] = 66.1–73.6) at baseline to 72.5% (IQR = 70.7–74.4) after a 1-month treatment (*p*_corr_ = 0.04). In the entire cohort, the change in its methylation level was found to be related to weight change, as shown in Fig. 1, although this association did not reach epigenome-wide statistical significance. The linear model predicted that a 2.8% difference in methylation level was associated with 0.85 kg of excess weight gain (95% CI 0.28–1.43, *p* = 0.004). Of note, this association remained significant when adjusting for white blood cell composition (0.78 kg of excess weight gain (95% CI 0.19–0.59, *p* = 0.01) when adjusting for estimated cell subtype variation and 0.86 kg of excess weight gain (95% CI 0.08–1.65, *p* = 0.03) when adjusting for measured neutrophil variation in the restricted sample).

### Epigenome-wide association analyses (EWAS) with metabolic phenotypes in PsyMetab and investigation of causality by Mendelian randomization

Associations between CpG methylation sites and changes in metabolic outcomes did not reach epigenome-wide significance. The Q–Q plots were checked and showed the absence of inflation, as shown in the Additional file 1: Fig. S2, for the EWAS investigating the association between the change in methylation levels (T1–T0) and the increase in body weight. Among the top 10 most significant associations of each EWAS (6 metabolic traits evaluated with baseline methylation and 1-month methylation change: 120 sites selected), only seven methylation loci were associated with a minimum of two meQTLs, enabling the estimation of a causal effect through MR (Table 3). One of the tested associations, cg11622362 (on chromosome 11), located close to the transcription start site of the *APIP* gene and opposite the *PDHX* gene body, revealed a significant effect. Namely, a higher methylation level was shown to causally relate to higher glucose levels (*p* = 10^–4^).

**Table 3:** Mendelian randomization analysis results of causal CpG sites for metabolic traits, selected among the top ten EWAS most significant hits

Exposure	MR results^a^					EWAS results^b^				CHR	CpG position in Genome Build 37	Reference gene^c^	Location of CpG related to gene^c^	Relation to CpG Island^d^
	Outcome	N SNPs	Beta	SE	*p*-value	CpG data	Beta	SE	*p*-value					
*cg11622362*	Glucose	2	0.019	0.005	**0.0001**	T0	− 1.05	0.22	2.96 × 10^–5^	11	34,938,112	APIP*	TSS200	Island
*cg09137125*	Glucose	2	− 0.003	0.007	0.63	T0	− 0.93	0.19	3.51 × 10^–5^	10	7,486,981			
*cg26295559*	Glucose	4	0.003	0.004	0.55	T0-T1	− 2.40	0.46	1.02 × 10^–5^	7	158,550,318	*ESYT2*	Body	
*cg09694300*	HDL	2	− 0.025	0.019	0.18	T0	− 1.25	0.28	5.58 × 10^–5^	3	159,429,667	*SCHIP1*	Body	
*cg23700124*	LDL	2	0.001	0.005	0.86	T0	− 0.69	0.14	1.15 × 10^–5^	4	123,843,736	SPATA5*	TSS1500	Island
*cg03803210*	TG	3	0.001	0.005	0.87	T0-T1	1.93	0.34	8.93 × 10^–7^	8	17,438,816	*PDGFRL*	Body	S_Shelf
*cg08387293*	TG	2	0.002	0.008	0.82	T0-T1	− 3.22	0.58	1.13 × 10^–6^	1	984,383	*AGRN*	Body	Island

*CHR* chromosome, *EWAS* epigenome-wide association study, *HDL* high density lipoprotein cholesterol, *LDL* low-density lipoprotein cholesterol, *MR* Mendelian randomization, *SE* standard error, *SNPs* single nucleotide polymorphisms, *TG* triglycerides

^a^Two-sample MR analyses using the inverse-variance weighted method were performed. Summary statistics for instrument-exposure association (n = 3,841) were retrieved from Bonder et al. [40], while instrument-outcome association statistics were derived from GWAS performed in the UKBiobank (http://www.nealelab.is/uk-biobank; *n* = 314′916 for glucose, *n* = 315′133 for HDL, *n* = 343′621 for LDL, *n* = 343,992 for TG). Significant *p*-values (< 0.05) are indicated in bold

^b^The results of the EWAS using methylation *M*-values are displayed. For the first line, the magnitude of the effect in *β*-value is to be interpreted as follows: a difference of 1.4% in baseline methylation level (corresponding to baseline methylation IQR in PsyMetab sample) is associated with a 0.39 mM (SE = 0.07) smaller glucose increase. No result reached statistical significance

^c^Reference genes for the methylation sites, and gene regions where the CpGs are located according to the UCSC database. Empty fields indicate an intergenic location

*Specifies there exists > 1 gene or gene transcript at this location. TSS200 = 0–200 bases upstream of the transcriptional start site (TSS); TSS1500 = 200–1500 bases upstream of the TSS; Body = between the ATG and stop codon, irrespective of the presence of introns, exons, TSS, or promoters

^d^The relation to a putative nearby CpG island, according to the UCSC database, is given. S_Shelf = 2–4 kb downstream (3′) of CpG island

In the psychiatric cohort, its baseline methylation level (median = 3.8%, IQR = 3.0–4.4) was negatively associated with a 1-month glucose change. Indeed, the model estimated a difference of 1.4% (IQR) in baseline methylation level to be associated with a 0.39 mM smaller glucose increase (95% CI 0.24–0.55), although this association did not reach epigenome-wide significance (*p* = 2.96 × 10^–5^). Of note, this association remained similar when adjusting for white blood cell composition (0.40 mM smaller glucose increase (95% CI 0.24–0.57, *p* = 1.91 × 10^–5^) when adjusting for estimated cell subtype variation and 0.21 mM smaller glucose increase (95% CI − 0.21 to 0.63, *p* = 0.3) when adjusting for measured neutrophil variation in the restricted sample).

### Validation of previous findings

Previous findings concerning *AKT2* and *FAR2* genes could be confirmed whereas results on *CDH22* gene could not be validated (Additional file 1: Table S2).

An association between *AKT2* methylation and glucose was indeed observed in our cohort. The median methylation level of the averaged 5 loci considered decreased from 3.4% (IQR = 2.9–3.8) at baseline to 3.3% (IQR = 2.3–4.2) after a 1-month treatment. The linear model predicted that a decrease of 0.1% in methylation level was associated with a 0.04 mM (SE = 0.02) reduction in glucose change (*p* = 0.02). However, no association between the baseline methylation level and glucose change was found.

The EWAS hit located in *FAR2* was also associated with glucose in our sample. Its baseline methylation level (median = 3.0%, IQR = 2.0–4.3), but not the change in methylation, was negatively associated with a 1-month glucose change. Indeed, the linear model estimated a difference of 2.3% (IQR) in baseline methylation level to be associated with a 0.43 mM (SE = 0.16) smaller glucose increase (*p* = 0.01).

The EWAS hit located in *CDH22* was not associated with MetS in our sample. Indeed, the logistic models did not reveal any significant association between the development of MetS and baseline methylation or change in methylation (*p* > 0.14 for all).

### Hypothesis-driven analyses

The three hypothesis-driven analyses did not uncover significant associations and only some trends were revealed. The results are detailed in the Additional file 1: Tables S3, S4 and S5.

## Discussion

### Global methylation changes

This study provided insights into a putative effect of psychotropic treatment on methylation as a mechanism leading to metabolic dysregulations. It assessed rapid changes in methylation following treatment initiation, and a global increase in methylation level was observed. This univariate analysis further showed that patients who gained ≥ 5% of their initial weight experienced a smaller 1-month methylation change than patients whose weight remained stable. This last observation shows that the increase in methylation levels likely occurs secondary to treatment and is not (at least not exclusively) a consequence of weight increase.

Nevertheless, it is challenging to distinguish medication-specific effects on DNA methylation from effects mediated by weight gain. For this purpose, future studies should include control groups of psychiatric patients with and without weight gain, but free from antipsychotics and compare the difference in the change in methylation occurring within one month. Unfortunately, such control groups were not available in the present study, and it is thus not possible to delineate the effect of the treatment, of weight increase and that of the natural course of disease. Most studies integrating control groups to date performed cross-sectional analyses, and the few researchers who have already evaluated longitudinal methylation changes pre- and post- psychotropic drug therapy only included baseline comparisons with control groups [8, 14, 43].

### Epigenome-wide methylation changes and association with weight change

Independent of weight increase, 52 methylation loci were shown to be significantly modified following the start of treatment. Most of these sites were located within a protein coding gene sequence and displayed an increase in methylation levels. The function of intragenic methylation is still largely unknown, but recent evidence supports a role of these methylation patterns in the regulation of alternative splicing [44–46]. In addition, a decrease in methylation was measured in three sites (cg24357026, cg27452651, cg02624558), found within CpG islands associated with promoters. As hypomethylation of CpG islands often correlates with active transcription of nearby genes [47, 48], the observed change in methylation may induce an increase in the expression of Zinc Finger Protein 146 (*ZNF146*), ALG12 Alpha-1,6-Mannosyltransferase (*ALG12*) and Lysine Demethylase 5B (*KDM5B*) genes. Interestingly, *ZNF146* and *KDM5B* gene ontology annotations according to GeneCards [49], both include *DNA-binding transcription factor activity*, implying that their activation likely affects the regulation of many other genes.

Regarding changes in methylation related to metabolic side effects, we observed that individuals who underwent marked weight gain experienced a significant increase in methylation level at one specific site (cg12209987). Moreover, this methylation change remained associated with weight evolution in multivariate analysis performed in all patients. This methylation locus is located within an enhancer, according to the Ensembl database [50], 60 kb upstream of the pseudogene *PSMC1P5*. The increased methylation at this site has no currently known function but may contribute to the regulation of enhancer activity [51].

These last three cited methylation modifications, rather than having a direct phenotypic impact, likely influence downstream pathways. As this analysis was conducted shortly after treatment onset, only early changes were detected. The modifications observed might lead to the regulation of a broader set of genes. In future studies, it would be interesting to follow the evolution of the methylation profile over time, using additional blood samples and detect whether the observed changes remain stable over the course of treatment, continue to evolve in the same direction or return to baseline; and whether methylation of other genes would occur in a second step.

### Epigenome-wide association analyses (EWAS) with metabolic phenotypes in PsyMetab and investigation of causality by Mendelian randomization

The statistically nonsignificant results we obtained in EWAS analyses possibly result from a lack of statistical power, considering the expected small effect sizes of single variations in DNA methylation. Besides, we used a conservative cutoff for FWER controlling, possibly leading to a certain rate of false negatives. Given the heterogeneity of the psychiatric population included—broad age range, diverse diagnoses, different current and past psychotropic drugs with various medication history—and the known impact these variables may have on metabolic side effects [52], a larger sample size would be required to identify hits. This would allow to further characterize the common involved mechanisms, and enable powerful stratified analyses, with more homogeneous groups with respect to specific factors.

Nevertheless, among the strongest associations observed, the causal role of the cg11622362 methylation level on glycemia could be established through an MR analysis. Importantly, the direction of the association was not concordant with the MR results, as high methylation at this locus was causally associated with elevated plasma glucose levels, while the longitudinal data showed an association of high methylation with a lower 1-month increase in plasma glucose. One thus needs to be cautious with causal assumptions because adiposity was shown to determine the alterations in methylation at the majority of the previously identified BMI-associated CpG sites [15, 53, 54]. Nevertheless, this finding deserves further research as the cg11622362 methylation site is located opposite the *PDHX* gene body. This gene encodes a subunit of the pyruvate dehydrogenase complex, which enables the conversion of pyruvate to acetyl coenzyme A, thereby linking glycolysis to the Krebs cycle. In addition, 3 SNPs located on this gene have been associated with type II diabetes mellitus, fasting blood glucose and insulin measurements, as well as with insulin resistance (HOMA-B) in recent GWAS [55, 56]. The functional and clinical relevance of this finding is thus notable, and methylation at cg11622362 may indeed have downstream effects influencing the change in plasma glucose levels following psychotropic treatment.

### Validation of previous findings

Interestingly, we validated previous findings in this specific field. We indeed found a signal for the implication of *AKT2* methylation in glucose homeostasis and confirmed an association between the previously identified EWAS hit in *FAR2* and glucose. The direction of both analyses was concordant with the literature [22, 23]. The reason why these two associations did not reach genome-wide statistical significance can be explained by several factors. First, *AKT2* was initially analysed in skeletal muscle and not blood samples. It is conceivable that the signal is attenuated in this marker tissue, but it is still very interesting to observe a convergent result. Additionally, the association was evaluated between an average of 22 methylation loci and insulin resistance, while we only had the data for 5 methylation sites in this genomic region, giving a less precise estimation of the methylation pattern. Last, the associations for both genes were estimated with glucose change, which is less stringent to indicate an abnormality in glucose homeostasis than insulin resistance. The relevance of the methylation patterns of *AKT2* and *FAR2* could be confirmed in a slightly different context, which gives a promising character to further research on these two genes.

### Limitations

It is important to mention that methylation patterns are highly tissue—or even cell-type—specific [57, 58]. Although we controlled for cellular heterogeneity using principal components (with additional sensitivity analyses using estimated cell subtype proportions and cellular composition for a subset of patients), the putative effects of psychotropic medication on blood might not be identical to those occurring in target tissues involved in metabolic dysfunctions (i.e.: brain, adipose tissue, liver, etc.), as previously reported in in vitro models [59]. Whether methylation patterns observed in blood might be relevant in the biological process leading to metabolic side effects or whether they can only be used as biomarkers attesting to related changes in the less accessible tissues of interest still needs validation [60]. Besides, and as previously mentioned, our cohort was treated with a variety of psychotropic drugs. The results observed in this study reflect putative shared mechanisms leading to metabolic adverse effects, but there may also be individual drug-specific changes that cannot be revealed. In addition, there is no perfect way to categorize treatments in terms of propensity to induce weight gain. There indeed exists various meta-analysis ordering antipsychotics according to their risk of weight gain. While it is clear that olanzapine induces greater weight gain than aripiprazole, there is considerable overlap in the confidence intervals for the drugs in between, and depending on the methodologies and the articles considered, the drug order sometimes differs [2, 33, 35, 52, 61, 62]. Moreover, a lack of head-to-head comparisons with the other psychotropic drugs prevents to make indisputable drug categories. Last, we were not able to control for diet, somatic comorbidities, substance use, trauma and other factors known for influencing specific epigenetic sites as well as metabolic health. It is thus important to keep in mind that the changes observed after the introduction of a psychotropic drug may be modulated by the patient’s history.

## Conclusion

In summary, appreciable changes in methylation levels were observed following the prescription of psychotropic treatments, but their role in the onset of metabolic side effects remains to be fully elucidated. With an improved understanding of the mechanisms behind such side effects, epigenetic biomarkers may contribute to precision medicine in the future.

### Supplementary Information

**Additional file 1**. Appendix: Supplementary Methods and Results. Supplementary figures and tables.

## Data Availability

The datasets analysed during the current study are not publicly available due to the sensitivity of the human personal data involved, which requires specific precautions and limitations. Procedures and documents related to data and/or material sharing (research application form, data and / or the material transfer agreements) are available via the link: http://www.chuv.ch/cnp-psymetab. The datasets analysed during the current study and/or the biological material used are available on reasonable request via the centralized e-mail address: research.psymetab@chuv.ch.
