# Supplementary material for: DNA methylation may partly explain psychotropic drug-induced metabolic side effects: results from a prospective 1-month observational study
Source: Clin Epigenetics. 2024 Feb 28;16:36. doi: 10.1186/s13148-024-01648-4 (PMC10903022; doi:10.1186/s13148-024-01648-4)
Supplement: Supplementary file 1 — Additional file 1. Appendix: Supplementary Methods and Results. Supplementary figures and tables. [file 13148_2024_1648_MOESM1_ESM.docx]

**Online Supplement:**

**Appendix:** Supplementary Methods and Results

**Supplementary Figure 1**: Flow chart for patients’ selection process

**Supplementary Figure 2**: Q-Q plot for the EWAS investigating the association between the change in methylation levels (T1-T0) and the increase in body weight (lambda ~0.83)

**Supplementary Table 1**: Main characteristics of selected vs unselected patients

**Supplementary Table 2:** Validation results of previous findings

**Supplementary Table 3:** Associations between selected methylation sites (hypothesis 1: epigenetic susceptibility to adiposity and CVD) and 1-month weight change

**Supplementary Table 4:** Associations between selected methylation sites (hypothesis 2: genetic susceptibility to AIWG) and 1-month weight change

**Supplementary Table 5:** Associations between selected methylation sites (hypothesis 3: genetic susceptibility to high BMI) and 1-month weight change

**Appendix**

**METHODS**

*Study design*


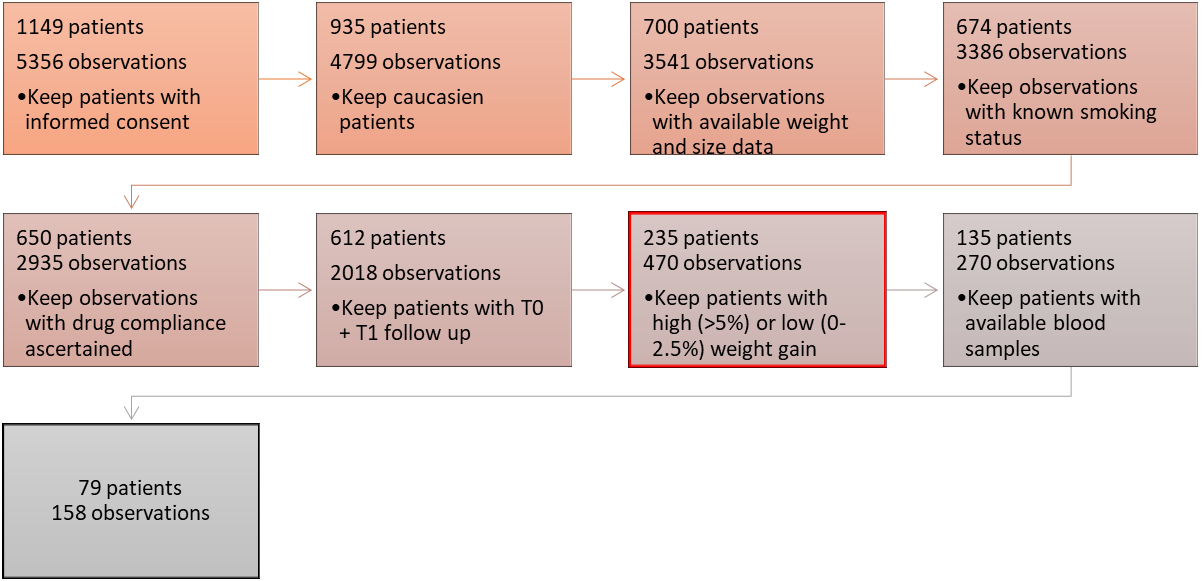

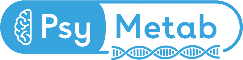


**Supplementary Figure 1**: Patients’ selection process from PsyMetab cohort

*Hypotheses-driven analyses*

In the first hypothesis-driven analysis, methylation sites previously reported to be linked to adiposity and CVD in the general population [1-3] were analyzed. Briefly, Wahl et al. identified 187 CpGs to be associated with BMI, and among them, 179 were present in our chip and were thus selected. Mendelssohn et al. identified 83 CpGs associated with BMI, with 38 of them already mentioned in Wahl et al. Out of the 45 CpGs remaining, 44 were available for analysis in our chip. As for the last study, Huan et al. identified 92 CpGs associated with various cardiometabolic outcomes and 85 of them were present in our chip. We selected only those associated with the metabolic phenotypes investigated in our study and ended up with 43 sites (including 4 on BMI).

The second approach was based on pharmacogenetic evidence of antipsychotic induced weight gain and aimed to explore whether the genetic susceptibility to metabolic side effects could be mediated by epigenetics. SNPs that were shown to be significantly associated with antipsychotic-induced weight gain (AIWG) in a recent meta-analysis were selected [4], and loci whose methylation levels were associated with these SNPs in *cis* [5] were analyzed.

Eventually, a similar hypothesis was followed, assumed on the genetic susceptibility to high BMI. First, SNPs associated with BMI in the general population, identified through GWAS [6], were selected. CpGs sites whose methylation levels were associated with these SNPs [5] were then analyzed. Finally, a transcriptome-wide summary statistics-based MR approach (TWMR), combining GWAS and expression quantitative traits loci (eQTLs) data, was applied to identify genes whose expression is causally associated with BMI [7, 8] and CpGs sites located on / nearby those genes were then analyzed.

**RESULTS**

*Study design*

To ensure the selection criteria based on the weight gain profile (red circled box in the flow chart) did not introduce a bias and that the selected sample accurately represents the total cohort, the main characteristics of selected and unselected patients are compared in Supplementary Table 1. No major difference is observable between the unselected and selected sample apart from the weight and metabolic data as expected.

*Epigenome-wide association analyses (EWAS) with metabolic phenotypes in PsyMetab*

All Q-Q plots were flat, consistent with the lack of statistically significant results. The Q-Q plot for the EWAS investigating the association between the change in methylation levels (T1-T0) and the increase in body weight (lambda ~0.83) is shown as an example in Supplementary Figure 2.


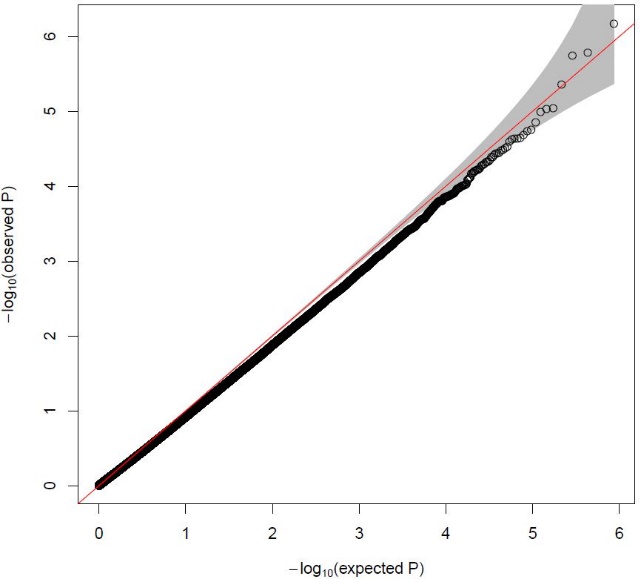


**Supplementary Figure 2**: Q-Q plot for the EWAS investigating the association between the change in methylation levels (T1-T0) and the increase in body weight (lambda ~0.83)

*Hypothesis-driven analyses*

The baseline and change in methylation level of 227 sites that had been linked to BMI in the general population [1-3] were tested for association with 1-month weight change, with no significant results. The 10 most significant associations are reported in Supplementary Table 2. The associations between the 39 methylation sites with a putative causal effect on CVD traits, selected from Huan et al [1], and the other metabolic phenotypes were also not statistically significant. Nevertheless, a trend was observed between increased 1-month methylation change at cg03649429 and decreased 1-month LDL cholesterol change (p_corr_ = 0.10, corrected for n=9 sites tested for association with LDL cholesterol change, data not shown). This methylation site is located in the body region of *CERT1* gene, upstream *POLK* gene on chromosome 5. In the general population, a higher methylation level at this locus was reported to induce a lower level of LDL cholesterol (p_corr_ = 1.9 × 10^−6^) [1].

Seven of the 13 SNPs related to AIWG, located on 6 genes, namely *SNAP25, INSIG2, ADRA2A, GNB3, BDNF and DRD2*, were associated with methylation level at 48 loci. Their baseline and change in methylation level were not significantly associated with 1-month weight change. The 10 most significant associations are reported in Supplementary Table 3.

The 88 independent BMI-associated SNPs identified in the UK Biobank [6] were associated with methylation levels of 466 loci. Alternatively, 754 methylation sites on the 38 genes whose expression is causally linked to BMI (via TWMR) were found. The baseline and change in methylation level at these sites were not significantly associated with 1-month weight change. The 10 most significant associations are reported in Supplementary Table 4. Only a trend was observed for a negative association between cg01264379 baseline methylation level and weight change (p_corr_ = 0.08).

**REFERENCE**

1. Huan, T., et al., *Genome-wide identification of DNA methylation QTLs in whole blood highlights pathways for cardiovascular disease.* Nature Communications, 2019. **10**(1): p. 4267.

2. Mendelson, M.M., et al., *Association of Body Mass Index with DNA Methylation and Gene Expression in Blood Cells and Relations to Cardiometabolic Disease: A Mendelian Randomization Approach.* PLoS Med, 2017. **14**(1): p. e1002215.

3. Wahl, S., et al., *Epigenome-wide association study of body mass index, and the adverse outcomes of adiposity.* Nature, 2016. **541**: p. 81.

4. Zhang, J.P., et al., *Pharmacogenetic Associations of Antipsychotic Drug-Related Weight Gain: A Systematic Review and Meta-analysis.* Schizophr Bull, 2016. **42**(6): p. 1418-1437.

5. Bonder, M.J., et al., *Disease variants alter transcription factor levels and methylation of their binding sites.* Nature Genetics, 2017. **49**(1): p. 131-138.

6. Locke, A.E., et al., *Genetic studies of body mass index yield new insights for obesity biology.* Nature, 2015. **518**(7538): p. 197-206.

7. Võsa, U., et al., *Unraveling the polygenic architecture of complex traits using blood eQTL metaanalysis.* 2018: p. 447367.

8. Porcu, E., et al., *Mendelian randomization integrating GWAS and eQTL data reveals genetic determinants of complex and clinical traits.* Nat Commun, 2019. **10**(1): p. 3300.

**Supplementary Table 1**: Main characteristics of selected vs unselected patients

|  | N | Total sample | Unselected sample (n=156) | Methylation sample (n=79) | p- value^2^ |
| --- | --- | --- | --- | --- | --- |
| *Age, median (range), y* | 235 | 38 (12-88) | 38.5 (12-88) | 37 (16-84) | 0.46 |
| *Men, n(%)* | 235 | 114 (48.5) | 74 (47.4) | 40 (50.6) | 0.64 |
| *Smoking, n(%)* | 235 | 105 (45.3) | 67 (43.0) | 40 (50.6) | 0.26 |
| *Main diagnosis, n(%)* | 217 |  | | | 0.11 |
| *Psychotic disorders (F20-F24; F28-F29)* |  | 90 (41.5) | 54 (39.1) | 36 (45.6) |  |
| *Schizoaffective disorders (F25)* |  | 20 (9.2) | 10 (7.3) | 10 (12.7) |  |
| *Bipolar disorders (F30-F31)* |  | 44 (20.3) | 26 (18.8) | 18 (22.8) |  |
| *Depressive disorders (F32-F33)* |  | 40 (18.4) | 32 (23.2) | 8 (10.1) |  |
| *Other* |  | 41 (10.6) | 16 (11.6) | 7 (8.9) |  |
| *Psychotropic treatment group, n(%)^3^* | 235 |  | | | 0.11 |
| *low risk of WG* |  | 48 (20.4) | 35 (22.4) | 13 (16.5) |  |
| *medium risk of WG* |  | 134 (53.2) | 92 (59.0) | 42 (53.2) |  |
| *high risk of WG* |  | 24 (30.4) | 29 (18.6) | 24 (30.4) |  |
| *BMI, median (range), kg/m^2^* | 235 |  | | |  |
| *Baseline* |  | 23.4 (15.2-41.7) | 23.6 (16.6-41.7) | 23.1 (15.2-37.5) | 0.06 |
| *First month* |  | 23.7 (16.9-43.5) | 23.6 (16.9-43.5) | 23.9 (17.0-39.5) | 0.58 |
| *p-value^4^* |  | **<10^-4^** | **0.01** | **<10^-4^** |  |
| *WG, median (range), %* | 235 | 1.6 (-14.5-23.0) | 1.3 (-14.5-13.5) | 2.4 (0-23.0) | **<10^-4^** |
| *Metabolic syndrome prevalence, n(%)^5^* | 148 |  | | |  |
| *Baseline* |  | 24 (16.2) | 21 (21.7) | 3 (5.9) | **0.01** |
| *First month* |  | 29 (19.6) | 21 (21.7) | 8 (15.7) | 0.39 |
| *p-value^4^* |  | 0.23 | 1.0 | 0.23 |  |

**Supplementary Table 2** Validation results of previous findings

| Exposure | Outcome | N | CpG data | Beta^1^ | SE | p-value | CHR | CpG position in Genome Build 37 | Reference gene^3^ | Location of cpg related to gene^3^ | Relation to CpG Island^4^ |
| --- | --- | --- | --- | --- | --- | --- | --- | --- | --- | --- | --- |
| *Candidate gene: AKT2^2^* | | | | | | | | | | | |
| Averaged methylation | Glucose | 25 | T0 | -0.60 | 0.70 | 0.4 | 19 | 40791288-40791462 | *AKT2* | TSS200 | Island |
|  |  |  | T0-T1 | 1.40 | 0.54 | **0.0165** |  |  |  |  |  |
| *EWAS hits^2^* | | | | | | | | | | | |
| cg10171063 | Glucose | 25 | T0 | -0.57 | 0.21 | **0.012** | 12 | 29302232 | *FAR2** | Body | Island |
|  |  |  | T0-T1 | 0.28 | 0.22 | 0.21 |  |  |  |  |  |
| cg04640913 | MetS | 52 | T0 | -0.14 | 2.63 | 0.96 | 20 | 44880515 | *CDH22** | TSS200 | S_Shore |
|  |  |  | T0-T1 | -51.5 | 35.2 | 0.14 |  |  |  |  |  |

CHR : chromosome; EWAS: epigenome-wide association study; MetS: metabolic syndrome; SE: standard error; N: number of patients included in the analyses

^1^The results of the analyses using methylation M-values are displayed. The magnitude of the significant effects in β-values are to be interpreted as follows: a T0-T1 difference of 0.1% in *AKT2* methylation level (corresponds to the median methylation decrease in PsyMetab sample) is associated with a 0.04 mM (SE = 0.02) smaller glucose increase; and a difference of 2.3% in baseline methylation level of cg10171063 (corresponds to baseline methylation IQR in PsyMetab sample) is associated with a 0.43 mM (SE = 0.16) smaller glucose increase.

^2^Genomic area previously analyzed in *AKT2*: Chr19:40791288-40791462, contains 22 CpGs sites averaged for analyses. 5 CpGs available in our data in this region: cg11812023, cg08874471, cg11794814, cg12299213, and cg15799967; pos: 40791312, 40791321, 40791331, 40791383, and 40791436, respectively. Previous findings from: gene candidate *AKT2*: Burghardt KJ et al, Pharmacotherapy, 2018. 38(4): p. 428-435; EWAS hit cg10171063: Burghardt KJ et al, Bipolar Disord, 2016. 18(5): p. 423-32; and EWAS hit cg04640913: Burghardt KJ et al, Int J Genomics, 2018. 2018: p. 8076397.

^3^Reference genes for the methylation sites, and gene regions where the CpGs are located according to the UCSC database. Empty fields indicate an intergenic location. * specify there exists >1 gene or gene transcript at this location. TSS200 = 0–200 bases upstream of the transcriptional start site (TSS); Body = Between the ATG and stop codon, irrespective of the presence of introns, exons, TSS, or promoters.

^4^The relation to a putative nearby CpG island, according to the UCSC database, is given. ; S_Shore = 0–2 kb downstream (3’) of CpG island.

| CpG site | CpG data | Beta | SE | p-value^1^ | CHR | CpG position^2^ | Reference gene^3^ | Location of CpG related to gene^3^ | Relation to CpG Island^4^ | Source^5^ |
| --- | --- | --- | --- | --- | --- | --- | --- | --- | --- | --- |
| *cg21429551* | T0 | -0.58 | 0.23 | 0.01 | 7 | 30635762 | *GARS* | Body | S_Shore | a, b |
| *cg19881557* | T0 | 1.03 | 0.45 | 0.03 | 14 | 20967426 |  |  |  | a |
| *cg13139542* | T0 | 0.70 | 0.31 | 0.03 | 2 | 8242815 |  |  |  | b |
| *cg07573872* | T0 | -0.70 | 0.33 | 0.04 | 19 | 1126342 | *SBNO2** | Body | S_Shelf | b |
| *cg05845030* | T0 | -0.90 | 0.48 | 0.07 | 12 | 91573247 | *DCN** | 5'UTR |  | a |
| *cg10513161* | T0 | 0.92 | 0.50 | 0.07 | 3 | 183705727 | *ABCC5** | Body |  | a |
| *cg03078551* | T0 | -0.80 | 0.44 | 0.07 | 17 | 41656298 | *ETV4* | Body |  | b |
| *cg18217136* | T0 | 0.99 | 0.55 | 0.08 | 20 | 36157651 | *BLCAP** | TSS1500 | S_Shore | a |
| *cg25649826* | T0 | 0.83 | 0.47 | 0.08 | 17 | 20938740 | *USP22* | Body |  | a, b |
| *cg17058475* | T0 | 0.36 | 0.21 | 0.10 | 11 | 68607737 | *CPT1A** | 5'UTR | N_Shore | b |
| *cg03078551* | T0-T1 | 1.79 | 0.58 | 0.003 | 17 | 41656298 | *ETV4* | Body |  | b |
| *cg01368219* | T0-T1 | -1.54 | 0.56 | 0.007 | 3 | 54999791 | *CACNA2D3** | Body |  | b |
| *cg13274938* | T0-T1 | -0.76 | 0.31 | 0.02 | 17 | 38493822 | *RARA** | Body | N_Shelf | a, b |
| *cg07573872* | T0-T1 | 1.76 | 0.81 | 0.03 | 19 | 1126342 | *SBNO2** | Body | S_Shelf | b |
| *cg00863378* | T0-T1 | -0.83 | 0.39 | 0.04 | 16 | 56549757 | *BBS2* | Body | N_Shelf | a |
| *cg21486834* | T0-T1 | -0.91 | 0.44 | 0.04 | 17 | 74477542 | *RHBDF2** | Body | S_Shelf | a |
| *cg01243823* | T0-T1 | -1.62 | 0.79 | 0.04 | 16 | 50732212 | *NOD2* | Body |  | a, b |
| *cg18217136* | T0-T1 | -1.14 | 0.59 | 0.06 | 20 | 36157651 | *BLCAP** | TSS1500 | S_Shore | a |
| *cg07471614* | T0-T1 | 0.81 | 0.44 | 0.07 | 8 | 125855152 |  |  |  | a |
| *cg01881899* | T0-T1 | 0.84 | 0.47 | 0.08 | 21 | 43652704 | *ABCG1** | Body |  | b |

**Supplementary Table 3** Associations between selected methylation sites (hypothesis 1: epigenetic susceptibility to adiposity and CVD) and 1-month weight change

CHR: chromosome; CVD: cardiovascular diseases

^1^Nominal p-values are reported. No association remained statistically significant after correcting for multiple testing (n=227 tested loci, selected from the literature^5^).

^2^CpG positions refer to Genome Research Consortium human genome build 37 (GRCh37)/UCSC human genome 19 (hg19)

^3^Reference genes for the methylation sites, and gene regions where the CpGs are located according to the UCSC database. Empty fields indicate an intergenic location. * specify there exists >1 gene or gene transcript at this location. TSS1500 = 200–1500 bases upstream of the transcriptional start site (TSS); 5'UTR = within the 5' untranslated region, between the TSS and the ATG start site; Body = Between the ATG and stop codon, irrespective of the presence of introns, exons, TSS, or promoters.

^4^The relation to a putative nearby CpG island, according to the UCSC database, is given. Shore = 0–2 kb from island; Shelf = 2–4 kb from island; N = upstream (5’) of CpG island; S = downstream (3’) of CpG island.

^5^Methylation sites selected from a: Wahl S et al, Nature, 2016. 541 :p.81; b : Mendelson MM et al, PLoS Med, 2017. 14(1) :p.e1002215; c: Huan T et al, Nature Communications, 2019. 10(1) :p.4267. NB : no sites from c were present in the top 10 most significant associations.

| CpG site | CpG data | Beta | SE | p-value^1^ | CHR | CpG position^2^ | Reference gene^3^ | Location of CpG related to gene^3^ | Relation to CpG Island^4^ | SNP^4^ | Gene^5^ |
| --- | --- | --- | --- | --- | --- | --- | --- | --- | --- | --- | --- |
| *cg19594606* | T0-T1 | 0.51 | 0.20 | 0.01 | 12 | 7023461 | *ENO2** | TSS200 | Island | rs5443 | GNBR |
| *cg04629424* | T0-T1 | -0.93 | 0.41 | 0.03 | 12 | 7020815 | *LRRC23** | Body | N_Shelf | rs5443 | GNBR |
| *cg08078216* | T0-T1 | -0.25 | 0.13 | 0.06 | 12 | 7053030 | *C12orf57* | TSS200 | N_Shore | rs5445 | GNBR |
| *cg08948964* | T0-T1 | 0.65 | 0.38 | 0.09 | 11 | 113285594 | *DRD2** | Body |  | rs6275 | DRD2 |
| *cg26269324* | T0-T1 | 0.20 | 0.12 | 0.10 | 12 | 6977084 | *TPI1** | Body | Island | rs5443 | GNBR |
| *cg12580156* | T0-T1 | 0.62 | 0.37 | 0.10 | 10 | 112588051 | *RBM20* | Body |  | rs1800544 | ADRA2A |
| *cg11367939* | T0-T1 | 0.47 | 0.30 | 0.12 | 10 | 112839302 | *ADRA2A** | 3'UTR | Island | rs1800544 | ADRA2A |
| *cg14346046* | T0-T1 | -0.69 | 0.48 | 0.16 | 11 | 27494792 | *LGR4* | TSS1500 | S_Shore | rs6265 | BDNF |
| *cg10635145* | T0-T1 | -1.00 | 0.75 | 0.19 | 11 | 27742435 | *BDNF** | TSS200 | N_Shore | rs6265 | BDNF |
| *cg03393638* | T0-T1 | 0.35 | 0.27 | 0.20 | 12 | 7046730 | *ATN1** | Body | Island | rs5443 | GNBR |
| *cg09360912* | T0 | -0.91 | 0.36 | 0.01 | 12 | 7000722 |  |  | Island | rs5443 | GNBR |
| *cg06684850* | T0 | -0.59 | 0.25 | 0.02 | 11 | 27742369 | *BDNF** | TSS200 | N_Shore | rs6265 | BDNF |
| *cg26269324* | T0 | -0.37 | 0.16 | 0.03 | 12 | 6977084 | *TPI1** | Body | Island | rs5443 | GNBR |
| *cg19594606* | T0 | -0.53 | 0.28 | 0.06 | 12 | 7023461 | *ENO2** | TSS200 | Island | rs5443 | GNBR |
| *cg03393638* | T0 | -0.57 | 0.31 | 0.07 | 12 | 7046730 | *ATN1** | Body | Island | rs5443 | GNBR |
| *cg10201141* | T0 | -0.64 | 0.38 | 0.10 | 12 | 7032793 | *ENO2** | 3'UTR |  | rs5443 | GNBR |
| *cg08078216* | T0 | 0.26 | 0.16 | 0.11 | 12 | 7053030 | *C12orf57* | TSS200 | N_Shore | rs5445 | GNBR |
| *cg16302655* | T0 | -0.39 | 0.24 | 0.11 | 11 | 113185879 | *TTC12* | 5'UTR | S_Shore | rs6275 | BDNF |
| *cg16158779* | T0 | -0.70 | 0.45 | 0.13 | 11 | 113258455 | *ANKK1* | TSS200 | Island | rs6275 | BDNF |
| *cg03425609* | T0 | -0.64 | 0.45 | 0.17 | 12 | 6952374 | *GNB3* | Body |  | rs5443 | GNBR |

**Supplementary Table 4** Associations between selected methylation sites (hypothesis 2: genetic susceptibility to AIWG) and 1-month weight change

AIWG: antipsychotic induced weight gain; CHR: chromosome; SNP: single nucleotide polymorphism

^1^Raw p-values are reported. No association remained statistically significant after correcting for multiple testing (n=48 tested associations).

^2^CpG positions refer to Genome Research Consortium human genome build 37 (GRCh37)/UCSC human genome 19 (hg19)

^3^Reference genes for the methylation sites, and gene regions where the CpGs are located according to the UCSC database. Empty fields indicate an intergenic location. * specify there exists >1 gene or gene transcript at this location. TSS200 = 0–200 bases upstream of the transcriptional start site (TSS); TSS1500 = 200–1500 bases upstream of the TSS; 5'UTR = Within the 5' untranslated region, between the TSS and the ATG start site; Body = Between the ATG and stop codon, irrespective of the presence of introns, exons, TSS, or promoters; 3'UTR = Between the stop codon and poly A signal.

^4^The relation to a putative nearby CpG island, according to the UCSC database, is given. Shore = 0–2 kb from island; Shelf = 2–4 kb from island; N = upstream (5’) of CpG island; S = downstream (3’) of CpG island.

^5^SNPs (from Genes) related to antipsychotic induced weight gain (Zhang JP et al, Schizophr Bull, 2016. 42(6): p. 1418-1437) and associated with methylation level at specific loci.

**Supplementary Table 5** Associations between selected methylation sites (hypothesis 3: genetic susceptibility to high BMI) and 1-month weight change

| CpG site | approach | CpG data | Beta | SE | p-value^1^ | CHR | CpG position^2^ | Reference gene^3^ | Location of CpG related to gene^3^ | Relation to CpG Island^4^ |
| --- | --- | --- | --- | --- | --- | --- | --- | --- | --- | --- |
| *cg09580214* | GWAS-SNPs | T0 | -0.48 | 0.15 | 0.002 | 11 | 47448534 | *PSMC3* | TSS1500 | S_Shore |
| *cg10614445* | GWAS-SNPs | T0 | 1.21 | 0.39 | 0.002 | 2 | 25526696 | *DNMT3A** | Body |  |
| *cg10648670* | GWAS-SNPs | T0 | -0.39 | 0.13 | 0.005 | 2 | 25439375 |  |  | S_Shore |
| *cg26378753* | GWAS-SNPs | T0 | 0.48 | 0.17 | 0.006 | 2 | 677591 | *TMEM18* | TSS200 | Island |
| *cg05223946* | GWAS-SNPs | T0 | 1.23 | 0.45 | 0.008 | 2 | 25056385 | *ADCY3* | Body |  |
| *cg14702927* | GWAS-SNPs | T0 | -0.75 | 0.29 | 0.01 | 19 | 46366445 | *SYMPK** | 5'UTR | Island |
| *cg24032752* | GWAS-SNPs | T0 | 0.49 | 0.19 | 0.01 | 3 | 50388670 | *NPRL2** | TSS200 | Island |
| *cg04633683* | GWAS-SNPs | T0 | -0.88 | 0.34 | 0.01 | 16 | 29837056 | *MVP** | 5'UTR |  |
| *cg19280586* | GWAS-SNPs | T0 | 0.77 | 0.30 | 0.01 | 15 | 68125197 | *LBXCOR1* | Body | N_Shore |
| *cg08972357* | GWAS-SNPs | T0 | 0.61 | 0.24 | 0.01 | 5 | 75009014 | *C5orf37** | TSS1500 | N_Shelf |
| *cg11774499* | GWAS-SNPs | T0-T1 | 2.73 | 0.96 | 0.006 | 19 | 46303628 | *RSPH6A* | Body | N_Shelf |
| *cg27237671* | GWAS-SNPs | T0-T1 | 1.86 | 0.66 | 0.006 | 2 | 676223 | *TMEM18* | Body | N_Shore |
| *cg22804499* | GWAS-SNPs | T0-T1 | 0.78 | 0.29 | 0.008 | 3 | 185632943 | *TRA2B* | 3'UTR |  |
| *cg08079166* | GWAS-SNPs | T0-T1 | -1.15 | 0.42 | 0.008 | 15 | 68083412 | *MAP2K5** | Body |  |
| *cg14726583* | GWAS-SNPs | T0-T1 | -1.61 | 0.66 | 0.02 | 6 | 40310514 |  |  |  |
| *cg06359938* | GWAS-SNPs | T0-T1 | -1.20 | 0.49 | 0.02 | 1 | 177891150 |  |  |  |
| *cg08253296* | GWAS-SNPs | T0-T1 | 1.02 | 0.42 | 0.02 | 5 | 74907592 |  |  | Island |
| *cg13322131* | GWAS-SNPs | T0-T1 | -2.69 | 1.11 | 0.02 | 6 | 50814305 | *TFAP2B* | 3'UTR | S_Shore |
| *cg26378753* | GWAS-SNPs | T0-T1 | -0.43 | 0.18 | 0.02 | 2 | 677591 | *TMEM18* | TSS200 | Island |
| *cg24235633* | GWAS-SNPs | T0-T1 | 0.89 | 0.39 | 0.02 | 16 | 29875177 | *CDIPT** | TSS1500 | S_Shore |
| *cg01264379* | **MR-Genes** | **T0** | **-0.65** | **0.16** | **0.0001** | **19** | **47617042** | ***ZC3H4*** | **TSS200** | **Island** |
| *cg22517087* | MR-Genes | T0 | -0.99 | 0.26 | 0.0004 | 12 | 49208544 |  |  | Island |
| *cg23704689* | MR-Genes | T0 | -0.61 | 0.17 | 0.0008 | 12 | 110906840 | *GPN3* | TSS1500 | Island |
| *cg10727340* | MR-Genes | T0 | 1.26 | 0.38 | 0.001 | 1 | 11199705 | *MTOR** | Body |  |
| *cg15068381* | MR-Genes | T0 | 1.04 | 0.33 | 0.002 | 3 | 185797716 | *ETV5* | Body |  |
| *cg04962026* | MR-Genes | T0 | 0.59 | 0.23 | 0.01 | 16 | 28509637 | *APOBR* | Body | S_Shore |
| *cg25641269* | MR-Genes | T0 | 0.52 | 0.20 | 0.01 | 3 | 185866557 | *DGKG** | 3'UTR |  |
| *cg01361881* | MR-Genes | T0 | 1.14 | 0.46 | 0.01 | 11 | 47267032 | *ACP** | Body | N_Shelf |
| *cg09988129* | MR-Genes | T0 | -0.39 | 0.16 | 0.02 | 7 | 74988866 | *STAG3L1** | 5'UTR | Island |
| *cg08248705* | MR-Genes | T0 | 1.14 | 0.46 | 0.02 | 1 | 11319406 | *MTOR* | Body | N_Shelf |
| *cg15068381* | MR-Genes | T0-T1 | -1.07 | 0.33 | 0.002 | 3 | 185797716 | *ETV5* | Body |  |
| *cg02359899* | MR-Genes | T0-T1 | -0.29 | 0.09 | 0.003 | 16 | 28503490 | *CLCN3** | 5'UTR | Island |
| *cg08239694* | MR-Genes | T0-T1 | 0.63 | 0.20 | 0.003 | 19 | 47636543 | *SAE1** | Body | S_Shelf |
| *cg18088359* | MR-Genes | T0-T1 | 0.90 | 0.29 | 0.003 | 12 | 49206892 | *CACNB3* | TSS1500 | N_Shore |
| *cg13076973* | MR-Genes | T0-T1 | 0.85 | 0.28 | 0.004 | 11 | 47612548 | *C1QTNF4* | 5'UTR | S_Shore |
| *cg10727340* | MR-Genes | T0-T1 | -0.98 | 0.34 | 0.005 | 1 | 11199705 | *MTOR** | Body |  |
| *cg18639436* | MR-Genes | T0-T1 | 0.90 | 0.31 | 0.005 | 3 | 185783611 | *ETV5** | Body |  |
| *cg25641269* | MR-Genes | T0-T1 | -0.43 | 0.15 | 0.007 | 3 | 185866557 | *DGKG** | 3'UTR |  |
| *cg07507257* | MR-Genes | T0-T1 | 0.84 | 0.30 | 0.008 | 18 | 21112105 | *NPC1* | 3'UTR |  |
| *cg06369573* | MR-Genes | T0-T1 | 0.98 | 0.41 | 0.02 | 19 | 19755321 | *GMIP* | TSS1500 | S_Shore |

CHR: chromosome; GWAS: genome-wide association study; MR: Mendelian randomization; SNP: single nucleotide polymorphism

^1^Raw p-values are reported. No association remained statistically significant after correcting for multiple testing (n=466 tested associations for the GWAS SNPs approach; n=754 tested associations for the MR-Genes approach). A trend (indicated in bold) was however present for the association between cg01264379 baseline methylation level and weight change (p_corr_ = 0.08).

^2^CpG positions refer to Genome Research Consortium human genome build 37 (GRCh37)/UCSC human genome 19 (hg19)

^3^Reference genes for the methylation sites, and gene regions where the CpGs are located according to the UCSC database. Empty fields indicate an intergenic location. * specify there exists >1 gene or gene transcript at this location. TSS200 = 0–200 bases upstream of the transcriptional start site (TSS); TSS1500 = 200–1500 bases upstream of the TSS; 5'UTR = within the 5' untranslated region, between the TSS and the ATG start site; Body = Between the ATG and stop codon, irrespective of the presence of introns, exons, TSS, or promoters; 3'UTR = between the stop codon and poly A signal.

^4^The relation to a putative nearby CpG island, according to the UCSC database, is given. Shore = 0–2 kb from island; Shelf = 2–4 kb from island; N = upstream (5’) of CpG island; S = downstream (3’) of CpG island.
